# Range of L5 LDL levels in healthy adults and L5’s predictive power in patients with hyperlipidemia or coronary artery disease

**DOI:** 10.1038/s41598-018-30243-w

**Published:** 2018-08-08

**Authors:** Chih-Sheng Chu, Hua-Chen Chan, Ming-Hsien Tsai, Nicole Stancel, Hsiang-Chun Lee, Kai-Hung Cheng, Yi-Ching Tung, Hsiu-Chuan Chan, Chung-Ya Wang, Shyi-Jang Shin, Wen-Ter Lai, Chao-Yuh Yang, Richard A. Dixon, Chu-Huang Chen, Liang-Yin Ke

**Affiliations:** 1 Lipid Science and Aging Research Center, Kaohsiung Medical University (KMU), Kaohsiung, Taiwan; 2 Center for Lipid Biosciences, KMU Hospital, KMU, Kaohsiung, Taiwan; 3 Department of Internal Medicine, KMU Hospital, KMU, Kaohsiung, Taiwan; 4 Vascular and Medicinal Research, Texas Heart Institute, Houston, TX USA; 5 Department of Public Health and Environmental Medicine, KMU, Kaohsiung, Taiwan; 6 Department of Medicine, Baylor College of Medicine, Houston, Texas USA; 7 Department of Molecular Cardiology, Texas Heart Institute, Houston, TX USA; 8 New York Heart Research Foundation, Mineola, NY USA; 9 Department of Medical Laboratory Science and Biotechnology, KMU, Kaohsiung, Taiwan

## Abstract

Electronegative L5 low-density lipoprotein (LDL) level may be a useful biomarker for predicting cardiovascular disease. We determined the range of plasma L5 levels in healthy adults (n = 35) and examined the power of L5 levels to differentiate patients with coronary artery disease (CAD; n = 40) or patients with hyperlipidemia (HLP) without evidence of CAD (n = 35) from healthy adults. The percent L5 in total LDL (L5%) was quantified by using fast-protein liquid chromatography with an anion-exchange column. Receiver operating characteristic curve analysis was performed to determine cut-off values for L5 levels. The mean L5% and plasma concentration of L5 (ie, [L5]) were significantly higher in patients with HLP or CAD than in healthy adults (*P* < 0.001). The ranges of L5% and [L5] in healthy adults were determined to be <1.6% and <1.7 mg/dL, respectively. In individuals with L5% >1.6%, the odds ratio was 9.636 for HLP or CAD. In individuals with [L5] >1.7 mg/dL, the odds ratio was 17.684 for HLP or CAD. The power of L5% or [L5] to differentiate patients with HLP or CAD from healthy adults was superior to that of the LDL/high-density lipoprotein ratio. The ranges of L5% and [L5] in healthy adults determined here may be clinically useful in preventing and treating cardiovascular disease.

## Introduction

Low-density lipoprotein-cholesterol (LDL-C) level has been globally recognized as a primary measure of atherosclerotic risk and is a therapeutic target for the prevention and treatment of cardiovascular disease^1,2^. In addition, the ratio of LDL to high-density lipoprotein cholesterol (HDL-C) (ie, the LDL/HDL ratio) has been considered an important marker for cardiovascular risk^3^. However, LDL-C levels have failed to predict the onset of cardiovascular disease in some cases^4,5^, and methods of measuring LDL-C have been shown to have analytical limitations^6^, indicating the need for an alternative, clinically useful biomarker of cardiovascular risk.

For more than two decades, an increased level of electronegative LDL in human plasma has been shown to be highly correlated to atherosclerosis and cardiovascular disease and has been implicated in the pathogenesis of these diseases^7–10^. More recently, electronegative LDL has been studied for its potential as a novel biomarker of cardiovascular risk^11,12^. The concept of electronegative LDL was first reported by Hoff and Gotto in 1979^13^. In 1988, Avogaro and colleagues isolated electronegative LDL [LDL(−)] from human LDL by using fast-protein liquid chromatography (FPLC) with an ion-exchange column^14^. Later in 2003, Yang and Chen used a modified version of that technique to further divide plasma LDL into 5 subfractions, L1-L5, according to charge^15,16^. With the use of a sequential separation technique, the most negatively charged LDL subfraction (ie, L5) and the least negatively charged LDL subfraction (ie, L1) are distinctly isolated from the transitional subfractions of human LDL (ie, L2-L4). Whereas LDL subfractions L1-L4 have no known harmful effects, L5 has various atherogenic effects *in vitro* and *in vivo*^7,11,16–19^ and has been identified as a novel cardiometabolic risk factor^11^. In addition, plasma levels of L5 have been shown to be elevated in patients with increased cardiovascular risk, including patients with hypercholesterolemia^16^, type II diabetes^20^, ST-elevation myocardial infarction (STEMI)^17^, and ischemic stroke^19^. Furthermore, the concentrations of LDL(−) and L5 in human plasma have been highly correlated to the severity of coronary artery disease (CAD)^21^.

Several studies have indicated that L5 may play a critical role in the pathology of STEMI and ischemic stroke^17,19^. L5 from STEMI patients increased adenosine 5′-diphosphate-stimulated platelet aggregation, platelet P-selectin expression, and GP IIb/IIIa activation and induced tissue factor expression, P-selectin expression, and apoptosis in endothelial cells^17^. In a mouse model in which thrombosis was induced in the middle cerebral artery, L5 was thrombogenic, whereas the genetic deficiency of L5’s receptor lectin-like oxidized LDL receptor-1 (LOX-1) had an inhibitory effect^19,22^. Thus, it has been suggested that the thrombophilic state created by L5 may cause a domino effect and produce an occlusive coronary artery thrombosis resulting in STEMI^23^.

Because LDL(−) and L5 circulate in human plasma, they have also been studied for their harmful effects in patients with chronic kidney disease^24^, patients undergoing dialysis^25^, and patients with systemic inflammation^26,27^. Furthermore, treatment with statins^18,28,29^ or sesamol^30^ has been shown to decrease the plasma level of LDL(−) or L5, respectively, and attenuate their inflammatory and apoptotic effects.

The importance of L5 LDL in the pathogenesis of STEMI and ischemic stroke has been well established and positively evaluated by experts^19,22^. In this study, we sought to define a range for plasma L5 levels in healthy adults. In addition, we examined the power of L5 levels to differentiate patients with hyperlipidemia (HLP) or CAD from healthy adults.

## Results

### Patient characteristics and biochemical profiles

Patient characteristics and biochemical profiles are shown in Table 1. Mean fasting blood sugar level was significantly different among healthy adults (ie, controls), HLP, and CAD groups (*P* = 0.016) and was significantly higher in the HLP group than in the control group (*P* = 0.020). However, no significant difference in mean fasting blood sugar level was observed between CAD and control groups (*P* = 0.075) or between CAD and HLP groups (*P* = 0.99). Mean total cholesterol level was significantly higher in the HLP group than in the control or CAD groups (*P* < 0.001 for HLP vs. control group; *P* = 0.001 for HLP vs. CAD group) and was significantly different between the control and CAD groups (*P* = 0.013). Mean triglyceride level was significantly higher in the HLP and CAD groups than in the control group (*P* = 0.021 for CAD vs. control group; *P* < 0.001 for HLP vs. control group). Mean HDL-C level was similar among control, CAD, and HLP groups (*P* = 0.504). Mean LDL-C level was significantly higher in the HLP group than in the CAD or control group (*P* = 0.019 and *P* < 0.001, respectively). In addition, mean LDL-C level was significantly higher in the CAD group than in the control group (*P* = 0.021). The mean LDL/HDL ratio was significantly lower in the control group than in the CAD or HLP group (*P* = 0.012 and *P* < 0.001, respectively), but no significant difference was observed between CAD and HLP groups (*P* = 0.347).

**Table 1:** Patient characteristics and biochemical profiles.

Baseline characteristic	Control (n = 35)	HLP (n = 35)	CAD (n = 40)	*P* value*
Men:women	19:16	15:20	23:17	0.421
Age (yr)	39.8 ± 12.0	56.5 ± 10.4	61.5 ± 9.4	<0.001
Patient history				
Diabetes (%)	0 (0/35)	31 (11/35)	25 (10/40)	0.002
Hypertension (%)	0 (0/35)	66 (23/35)	68 (27/40)	<0.001
Dyslipidemia (%)	0 (0/35)	100 (35/35)	55 (22/40)	<0.001
Medications†				
Antiplatelet drugs (%)	0 (0/35)	3 (1/35)	18 (7/40)	0.007
Statin (%)	0 (0/35)	3 (1/35)	20 (8/40)	0.003
Treatment for high BP (%)	0 (0/35)	0 (0/35)	13 (5/40)	<0.001
Glu AC (mg/dL)	96.3 ± 17.4	115.0 ± 35.6	111.2 ± 28.8	0.016
Lipid profile				
T-CHOL (mg/dL)	173.4 ± 32.8	235.9 ± 36.6	200.7 ± 48.9	<0.001
TG (mg/dL)	79.7 ± 56.1	164.5 ± 90.6	133.1 ± 96.6	<0.001
HDL-C	54.4 ± 14.0	53.1 ± 16.4	50.2 ± 17.6	0.504
LDL-C	103.3 ± 27.6	146.0 ± 34.9	124.5 ± 36.3	<0.001
LDL/HDL	2.02 ± 0.79	2.95 ± 1.00	2.63 ± 0.84	<0.001
L1%	86.6 ± 7.2	69.9 ± 22.7	60.8 ± 24.1	0.001
L2%	5.3 ± 4.7	10.5 ± 9.0	13.5 ± 9.7	0.010
L3%	6.5 ± 3.3	16.3 ± 14.6	20.0 ± 16.7	0.011
L4%	0.4 ± 0.5	1.1 ± 1.4	2.0 ± 2.8	0.027
L5%	1.30 ± 0.66	2.28 ± 1.32	3.65 ± 2.32	<0.001
[L5]^‡^ (mg/dL)	1.31 ± 0.70	3.24 ± 2.00	4.33 ± 2.48	<0.001

^*^P-value for the analysis of variance or chi-square test.

^†^Medications taken within 3 months before study enrollment, including anti-platelet drugs such as aspirin and clopidogrel; statins such as atorvastatin and rosuvastatin; and high blood pressure treatment such as amlodipine and amlodine/benazepril.

^‡^[L5], the concentration of L5, calculated as L5% × LDL-C (mg/dL).

BP = Blood pressure; CAD = (stable) coronary artery disease; Glu AC = fasting blood sugar; HDL-C = high-density lipoprotein cholesterol; HLP = hyperlipidemia without coronary artery disease; LDL-C = low-density lipoprotein cholesterol; L5% = L5 percentage; T-CHOL = total cholesterol; TG = triglyceride.

### The elevation of plasma L5 percentage and L5 concentration in HLP and CAD patients vs. controls

**Figure 1:**
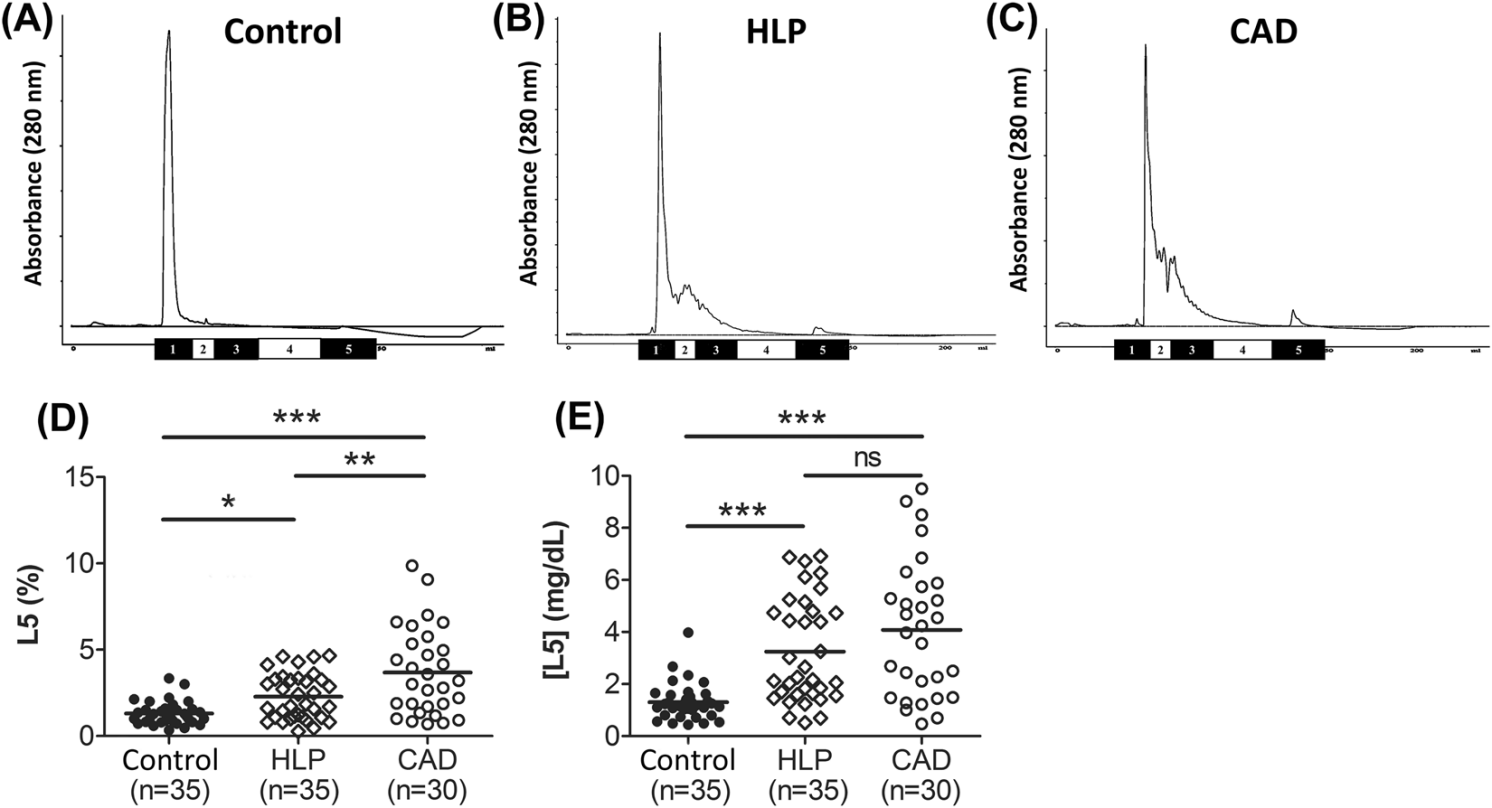
L5 plasma concentrations in healthy controls and patients with HLP or CAD. FPLC analysis of LDL showing the content of each subfraction (L1-L5) in (**A**) healthy adults with no cardiovascular risk factors, (**B**) patients with HLP without CAD, and (**C**) patients with stable CAD. (**D**) The L5% and (**E**) [L5] values for each individual are plotted. ^*^P < 0.05, ^**^P < 0.01, and ^***^P < 0.001, determined by using ANOVA with the Bonferroni adjustment. Lines represent the group means. ns, nonsignificant (*P* > 0.05).

**Figure 2:**
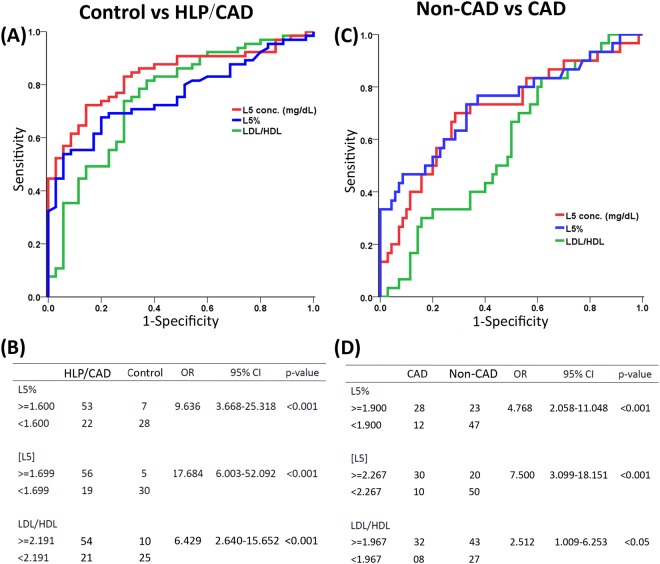
Receiver operating characteristic curve analysis for L5%, [L5], and the LDL/HDL ratio. (**A**) Comparison of the control vs. the HLP/CAD group: the area under the curve was 0.776 for L5%, 0.849 for [L5], and 0.745 for the LDL/HDL ratio. (**B**) Odds ratio of L5%, [L5], and the LDL/HDL ratio. (**C**) Comparison of the non-CAD vs. CAD groups: area under the curve was 0.752 for L5%, 0.741 for [L5], and 0.563 for the LDL/HDL ratio. (**D**) Odds ratio of L5%, [L5], and the LDL/HDL ratio.

Percentages of L1-L5 in total LDL (ie, L1%–L5%) were measured by using FPLC and were compared among controls and patients with HLP or CAD (Table 1 and Fig. 1). The mean L1% was significantly different among control, HLP, and CAD groups (ANOVA *P* = 0.001; Table 1). Bonferroni post hoc analysis revealed that the mean L1% was significantly decreased in the HLP group (*P* = 0.044) and the CAD group (*P* = 0.001) compared with the control group. The mean L5% was also significantly different among control, HLP, and CAD groups (*P* < 0.001). Bonferroni post hoc analysis revealed that the mean L5% was significantly elevated in the HLP group (*P* = 0.042; Figs 1B, 2B, and Table 1) and CAD group (*P* < 0.001; Fig. 1C,D, and Table 1) compared with the control group. Notably, the mean L5% of CAD patients was significantly different from that of HLP patients (*P* = 0.001; Fig. 1D). Furthermore, the mean plasma L5 concentration ([L5]), defined as L5% multiplied by LDL-C, was significantly different among control, HLP, and CAD groups (*P* < 0.001; Fig. 1E and Table 1). The mean [L5] in the CAD or HLP group was significantly higher than that of the control group (*P* < 0.001 and *P* < 0.001, respectively). In addition, the mean [L5] of the CAD group was significantly higher than that of the HLP group (*P* = 0.047; Fig. 1E).

### Comparison of lipid profiles between healthy controls and HLP/CAD patients

Hyperlipidemia is an important risk factor for CAD; therefore, we combined these two groups into one (ie, HLP/CAD) for further analysis. The comparison of patient characteristics and biochemical profiles between the control and HLP/CAD groups showed no difference in mean HDL-C level (*P* = 0.383). However, the mean age, LDL/HDL, and levels of fasting glucose, total cholesterol, triglyceride, LDL-C, L5%, and [L5] were significantly higher in the HLP/CAD group than in the control group (Table 2). When we adjusted for these factors using multivariate analysis, we found that the mean age and levels of total cholesterol, triglyceride, and L5% remained significantly higher in the HLP/CAD group than in the control group (*P* = 0.005, 0.009, 0.010, and 0.032, respectively), whereas the mean LDL-C and HDL-C levels were not significantly different between the HLP/CAD group and the control group. [L5] was excluded from the multivariate analysis because it is derived from the other factors L5% and LDL-C.

**Table 2:** Multivariate analysis of factors associated with HLP/CAD.

Baseline characteristic	Control (n = 35)	HLP/CAD (n = 75)	Univariate *P* value*	Multivariate analysis^§^	
				OR (95% CI)	*P* value
Men:women	19:16	38:37	0.882	—	NS^||^
Age (yr)	39.8 ± 12.0	59.2 ± 10.2	<0.001	1.438 (1.117–1.850)	0.005
Patient history					
Diabetes (%)	0 (0/35)	28 (21/75)	<0.001		
Hypertension (%)	0 (0/35)	67 (50/75)	<0.001		
Dyslipidemia (%)	0 (0/35)	76 (57/75)	<0.001		
Medications†					
Antiplatelet drugs (%)	0 (0/35)	11 (8/75)	0.053		
Statin (%)	0 (0/35)	12 (9/75)	0.055		
Treatment for high BP (%)	0 (0/35)	7 (5/75)	0.176		
Glu AC (mg/dL)	96.3 ± 17.4	113.0 ± 32.0	0.005	—	NS
Lipid profile					
T-CHO (mg/dL)	173.4 ± 32.8	217.2 ± 46.8	<0.001	1.091 (1.022–1.165)	0.009
TG (mg/dL)	79.7 ± 56.1	147.7 ± 94.5	<0.001	1.032 (1.008–1.0–7)	0.010
HDL-C (mg/dL)	54.4 ± 14.0	51.5 ± 17.0	0.383	—	NS
LDL-C (mg/dL)	103.3 ± 27.6	134.6 ± 37.0	<0.001	—	NS
LDL/HDL	2.02 ± 0.79	2.78 ± 0.93	<0.001	—	NS
L5%	1.30 ± 0.66	3.01 ± 2.03	<0.001	11.32 (1.24–103.40)	0.032
[L5]^‡^ (mg/dL)	1.30 ± 0.70	3.82 ± 2.32	<0.001	—	^§^

^*^P-value for Student *t*-test or chi-square test.

^†^Medications taken within 3 months before study enrollment, including anti-platelet drugs such as aspirin and clopidogrel; statins such as atorvastatin and rosuvastatin; and high blood pressure treatment such as amlodipine and amlodine/benazepril.

^‡^[L5], the concentration of L5, calculated as L5% × LDL-C (mg/dL).

^§^Multivariate analysis: the logistic regression analysis was with L5%; [L5] was excluded because it is derived from L5% × LDL-C (mg/dL).

^||^NS, not significant.

BP = blood pressure; CAD = (stable) coronary artery disease; Glu AC = fasting blood sugar; HDL-C = high-density lipoprotein cholesterol; HLP = hyperlipidemia without CAD; LDL-C = low-density lipoprotein cholesterol; L5% = L5 percentage in total LDL; T-CHOL = total cholesterol; TG = triglyceride.

### The ranges of L5% and [L5] in healthy adults and the predictive value of L5% and [L5] for HLP/CAD

Receiver operating characteristic (ROC) curve analysis was performed separately between the control and HLP/CAD groups to determine the ranges of L5% and [L5] in healthy adults, as well as the diagnostic predictive value of L5%, [L5], and the LDL/HDL ratio for HLP/CAD for HLP/CAD (Fig. 2). According to the results of our ROC curve analysis, the area under the curve was 0.776 for L5% (95% CI, 0.692–0.861; *P* < 0.001) and 0.849 for [L5] (95% CI, 0.778–0.920; *P* < 0.001), which is classified as having good accuracy for a diagnostic test. The area under the curve was 0.745 for the LDL/HDL ratio (95% CI, 0.644–0.846; *P* < 0.001). Thus, L5% and [L5] significantly predicted HLP/CAD (Fig. 2B).

According to the ROC curve of L5% for the HLP/CAD group, the best cut-off value (ie, the greatest sum of sensitivity and specificity-1) was 1.6%. Therefore, the range of L5% in healthy adults was determined to be less than 1.6%. The odds ratio of L5% greater than 1.6% for HLP/CAD was 9.636 (95% CI, 3.668–25.318; *P* < 0.001; Fig. 2B), indicating that individuals who have an L5% greater than 1.6% have a 9.6-fold increased chance of having HLP or CAD.

According to the ROC curve of [L5] for the HLP/CAD group, the best cut-off value was 1.7 mg/dL. Therefore, the range of [L5] in healthy adults was determined to be less than 1.7 mg/dL. The odds ratio of [L5] greater than 1.7 was 17.684 (95% CI, 6.003–52.092; *P* < 0.001; Fig. 2B), indicating that individuals who have an [L5] higher than 1.7 mg/dL have a 17.7-fold increased chance of having HLP or CAD. In contrast, the ROC curve of the LDL/HDL ratio for the HLP/CAD group showed that the best cut-off value was 2.2. The odds ratio of an LDL/HDL ratio greater than 2.2 was 6.429 (95% CI, 2.640–15.652; *P* < 0.001; Fig. 2B), indicating that individuals with an LDL/HDL ratio of 2.2 have a 6.4-fold increased chance of having HLP/CAD. Thus, our analyses show that L5% and [L5] had a higher predictive value of HLP/CAD than did the LDL/HDL ratio.

### L5% and [L5]: potentially useful biomarkers for predicting cardiovascular disease

Although hyperlipidemia is an important risk factor for CAD, CAD may occur independently of hyperlipidemia. In addition, L5% is an independent risk factor different from cholesterol or triglycerides. To account for these matters, we also examined the effect of L5% on CAD by combining data for the control and HLP groups (ie, the non-CAD group) and compared them with data for the CAD group (Table 3). No differences were observed in the mean levels of fasting blood sugar, total cholesterol, triglyceride, HDL-C, or LDL-C, or in the LDL/HDL ratio between the non-CAD and CAD groups. In contrast, the mean L5% and [L5] were significantly higher in the CAD group than in the non-CAD group (*P* < 0.001 respectively) (Table 3). ROC curve analysis was performed separately between the non-CAD and CAD groups to determine the diagnostic predictive value of L5%, [L5], and the LDL/HDL ratio for CAD (Fig. 2C). The predictive value of the LDL/HDL ratio was assessed alongside L5% and [L5] because it is a known risk factor for cardiovascular disease. According to the results of our ROC analysis between the non-CAD and CAD groups, an increase in L5% and [L5] was observed in the CAD group (Fig. 2C). The area under the curve was 0.753 for L5% (95% CI, 0.652–0.853; *P* < 0.001) and 0.741 for [L5] (95% CI, 0.641–0.842; *P* < 0.001). In contrast, the area under the curve was 0.563 for the LDL/HDL ratio (95% CI, 0.455–0.670; *P* = 0.277), which was not as significant as either L5% or [L5] for predicting CAD.

**Table 3:** Multivariate analysis of factors associated with CAD.

Baseline characteristic	Non-CAD (n = 70)	CAD (n = 40)	Univariate *P*-value*	Multivariate analysis§	
				OR (95% CI)	*P* value
Men:women	34:36	23:17	0.482	0.152 (0.037–0.613)	0.008
Age (yr)	48.1 ± 13.96	61.6 ± 9.44	0.002	1.078 (1.016–1.143)	0.013
Patient history					
Diabetes (%)	16 (11/70)	25 (10/40)	0.347	—	NS^||^
Hypertension (%)	33 (23/70)	68 (27/40)	0.001	—	NS
Dyslipidemia (%)	50 (35/70)	55 (22/40)	0.759	—	NS
Medications†					
Antiplatelet drugs (%)	1.4 (1/70)	18 (7/40)	0.006	0.056 (0.005–0.684)	0.024
Statin (%)	1.4 (1/70)	20 (8/40)	0.002	—	NS
Treatment for high BP(%)	0 (0/70)	13 (5/40)	0.011	—	NS
Glu AC (mg/dL)	105.6 ± 29.40	111.2 ± 28.83	0.341	—	NS
Lipid profile					
T-CHO (mg/dL)	204.7 ± 46.72	200.7 ± 48.91	0.677	—	NS
TG (mg/dL)	122.1 ± 86.13	133.1 ± 96.58	0.540	—	NS
HDL-C (mg/dL)	53.8 ± 15.14	50.17 ± 17.64	0.262	—	NS
LDL-C (mg/dL)	124.7 ± 37.94	124.5 ± 36.28	0.986	—	NS
LDL/HDL	2.49 ± 1.00	2.63 ± 0.84	0.465	—	NS
L5%	1.79 ± 1.15	3.65 ± 2.32	< 0.001	2.441 (1.541–3.774)	<0.001
[L5]^‡^ (mg/dL)	2.27 ± 1.78	4.33 ± 2.48	< 0.001		^§^

^*^P-value for Student *t*-test or chi-square test.

^†^Medications taken within 3 months before study enrollment, including anti-platelet drugs such as aspirin and clopidogrel; statins such as atorvastatin and rosuvastatin; and high blood pressure treatment such as amlodipine and amlodine/benazepril.

^‡^[L5], the concentration of L5, calculated as L5% × LDL-C (mg/dL).

^§^Multivariate analysis: the logistic regression analysis was with L5%; [L5] was excluded because it is derived from L5% × LDL-C (mg/dL).

^||^NS, not significant.

BP = blood pressure; CAD = (stable) coronary artery disease; Glu AC = fasting blood sugar; HLP = hyperlipidemia without CAD; HDL-C = high-density lipoprotein cholesterol; LDL-C = low-density lipoprotein cholesterol; L5% = L5 percentage; T-CHOL = total cholesterol; TG = triglyceride.

According to the ROC curve of L5% for CAD, the best cut-off value was 1.9%. The odds ratio of L5% greater than 1.9% was 4.768 (95% CI, 2.058–11.048; *P* < 0.001) (Fig. 2D), indicating that individuals who have an L5% higher than 1.9% have 4.8-fold increased chance of having CAD, regardless of lipid profile. According to the ROC curve of [L5] for CAD, the best cut-off value was 2.3 mg/dL. The odds ratio of [L5] greater than 2.3 mg/dL was 7.500 (95% CI, 3.099–18.151; *P* < 0.001) (Fig. 2D), indicating that individuals who have an [L5] concentration higher than 2.3 mg/dL have a 7.5-fold increased chance of having CAD, regardless of lipid profile. Therefore, we determined the ranges of L5% and [L5] in CAD patients to be greater than 1.9% and greater than 2.3 mg/dL, respectively. According to the ROC curve of the LDL/HDL ratio for CAD, the best cut-off value was 2.0. The odds ratio of an LDL/HDL ratio greater than 2.0 was 2.512 (95% CI, 1.009–6.253; *P* = 0.034). These findings indicated that L5% and [L5] have better predictive values for CAD than does the LDL/HDL ratio.

## Discussion

In this study, we evaluated the L5% and [L5] in healthy adults, patients with hyperlipidemia without evidence of CAD, and patients with CAD. In addition, we defined the upper limits of the ranges for L5% (<1.6%) and [L5] (<1.7 mg/dL) in healthy adults. Moreover, we examined the power of L5% and [L5] to differentiate patients with HLP or CAD (ie, HLP/CAD) from healthy adults and found that L5% and [L5] are superior in this regard to the LDL/HDL ratio, previously shown to have a high predictive value of cardiovascular risk^31^. Because [L5] is derived from L5% and the total LDL concentration, [L5] alone can be used to determine cardiovascular risk in the clinical setting.

Because of the variability among methods used to isolate highly electronegative LDL, a normal range for L5 has not been previously established. The use of an FPLC machine equipped with an anion-exchange column is presently the most common method for detecting and quantifying either LDL(−) or L5 in human plasma. Several laboratories have reported the use of different anion-exchange columns^14,15,17,32,33^. However, the most notable difference among the reported laboratory procedures has been the NaCl gradient. With the use of a multistep linear gradient with NaCl concentrations between 0.15–0.20 M, which are representative of normal physiologic conditions, LDL subfractions L1 and L5 can be distinctly separated^15,17^. However, limitations of the current method used for quantifying L5 are that it is time consuming and requires a large blood sample. Therefore, an alternative, more rapid method is required for performing large-scale clinical trials and for quantifying L5 in the clinical setting.

Similar to diabetes and hypertension, dyslipidemia is a risk factor for cardiovascular disease^34,35^. Currently, the ratio of LDL/HDL is the prime index of cardiovascular disease risk^36^. The lowering of LDL-C levels is the main approach for the primary and secondary prevention of cardiovascular diseases^1^. However, in studies of STEMI and ischemic stroke patients in whom L5 levels were elevated, the LDL-C levels of these patients were found to be normal^17,19,37^. In addition, our findings indicated that [L5] and L5% are better than the LDL/HDL ratio as predictors for differentiating HLP or CAD patients from healthy individuals. Indeed, our ROC curve analysis indicated that [L5] and L5% had superior sensitivity and specificity for differentiating patients with HLP/CAD from healthy individuals. Thus, our findings support that electronegative L5 LDL level is an important biomarker for cardiovascular disease^11^.

Previous studies have shown that the most electronegative subfraction of LDL, L5, has atherogenic effects in various cell types in *vitro* and in *vivo*^7,8,10^, whereas the least electronegative subfraction, L1 (ie, native LDL), does not. In the present study, we found that L1% was significantly decreased in the HLP or CAD groups when compared with that in the control group (*P* = 0.001). LDL is important for delivering cholesterol to tissues daily. Unlike L5^38^, L1 undergoes LDL receptor–mediated endocytosis before it is hydrolyzed into amino acids and free cholesterol, which are used as a substrate for the biosynthesis of important organic compounds^39^. Our finding that L1% is decreased and that L5% is increased in the plasma of HLP or CAD patients further supports that the increased electronegativity of LDL—as measured by L5 levels—represents an important biomarker of cardiovascular risk.

In addition to determining the ranges for L5% and [L5] in healthy adults, we also performed analyses to determine the ranges for these L5 values in patients with CAD, which were >1.9% for L5% and >2.3 mg/dL for [L5]. These ranges were determined by comparing data between CAD patients and controls/HLP patients (non-CAD group). Our ROC curve analysis in these groups showed that L5% and [L5] have better sensitivity and specificity for predicting CAD. However, the ROC curve was not perfect, most likely because cardiovascular disease can be caused by a variety of factors such as an unhealthy diet, obesity, smoking, diabetes, and inflammation.

In conclusion, we have identified ranges of L5% and [L5] in healthy adults and have shown that these L5 values are significantly increased in patients with HLP or CAD. Although we acknowledge that sample size is an important limitation of this study, this was a pilot study that we plan to expand into a full-scale study in the future. Nonetheless, our findings support that circulating levels of electronegative L5 represent a powerful clinical biomarker that can be used to differentiate patients with HLP or CAD from healthy individuals, warranting its clinical use.

## Methods

### Study design and clinical diagnoses

This study was approved by the institutional review boards of Kaohsiung Medical University Hospital (KMUH) and Kaohsiung Municipal Ta-Tung Hospital (KMTTH) in Taiwan. From October 2010 to August 2012, 110 individuals were recruited for this study through KMUH or KMTTH. Informed consent was obtained from all study participants, and all procedures were performed according to the Declaration of Helsinki. All enrolled patients were seen by cardiologists at KMUH or KMTTH. Patients were categorized into one of three study groups: the CAD group, the hyperlipidemia (HLP) group without evidence of CAD, or the healthy control group. For patients in the CAD group, the diagnosis of CAD was made by using coronary arteriography to identify any vascular stenosis with >50% luminal narrowing (as determined by using quantitative coronary analysis) in the main coronary arteries or major branches. Patients in the CAD group may or may not have had hyperlipidemia because it was not used as a criterion. Patients in the HLP group had hyperlipidemia without evidence of CAD. The diagnosis of hyperlipidemia was made if cholesterol levels were higher than 200 mg/dL or triglyceride levels were higher than 150 mg/dL. Finally, healthy controls had no evidence of CAD or hyperlipidemia. According to these criteria, 40 patients were determined to have stable CAD, and 35 patients were determined to have hyperlipidemia without evidence of CAD. The control group comprised 35 healthy adults without known cardiovascular risk factors.

Blood samples were collected from participants before treatment. Clinical and medical histories from the 3 months that preceded case enrollment were recorded for each patient. Lipid parameters for all study participants were measured in the Department of Laboratory Medicine at KMUH (accredited by the College of American Pathologists) according to standard operating procedures and included total cholesterol, triglyceride, HDL-C, LDL-C, and fasting blood sugar.

### LDL isolation and L5 analysis

Whole blood samples (20 mL) were freshly collected and anticoagulated with 5 mM EDTA. The blood was immediately centrifuged at 3000 rpm for 10 minutes to remove red blood cells. To prevent contamination and protein degradation, 1% penicillin/streptomycin and Complete Protease Inhibitor Cocktail (Roche Diagnostics, Indianapolis, IN; 1 tablet/100 mL plasma) were added to plasma samples. EDTA (0.5 mg/mL) and N_2_ were also added to avoid sample oxidation during the entire process of preparation and preservation. LDL (density = 1.019–1.063) was then isolated by using sequential potassium bromide density centrifugation with a Beckman Optima L-100K ultracentrifuge (Beckman Coulter, Inc., Brea, CA) equipped with a Type 90 Ti fixed-angle rotor (Beckman Coulter). After dialysis with buffer A (0.02 M Tris-HCl, 0.5 mM EDTA at pH 8.0) 3 times for 24 hours each time, LDL samples were injected into an ÄKTA FPLC system (GE Healthcare Life Sciences, Pittsburgh, PA) equipped with an anion-exchange Uno-Q12 column. Using a multistep linear NaCl gradient, LDL was further divided into subfractions L1, L2, L3, L4, and L5 as previously described^15,38^. The effluent was monitored at 280 nm and protected from *ex vivo* oxidation with 5 mM EDTA. Each subfraction from healthy controls, patients with HLP, or patients with CAD was concentrated by using Centriprep® filters (YM-30; EMD Millipore Corp., Billerica, MA) and sterilized by passing through 0.22-μm filters. The isolated subfractions were N_2_-sealed and stored at 4 °C during sample characterization^40^.

### Statistical analysis

All data are presented as relative frequencies for discrete responses and as the mean ± standard deviation for continuous responses. Comparisons of 3 or more groups were performed by using analysis of variance (ANOVA). The Bonferroni post hoc method was used for multiple comparisons. If the initial ANOVA *P*-value was not significant, no further pairwise comparisons were performed. A Student *t* test and chi-square test were used to compare the difference between 2 groups (control and test group). A binary multivariate logistic regression model was used to estimate the adjusted odds ratio of the independent variable upon the dependent variable. ROC curve analysis was performed to determine the cut-off values for L5 plasma levels. Using these cut-off values, we defined the upper limits of the ranges for L5% and [L5] in healthy individuals and CAD patients. By comparing the healthy controls and patients with HLP or CAD (ie, control vs. HLP/CAD), we evaluated the ranges of L5% or [L5] in individuals without any cardiovascular risks or diseases. By comparing the non-CAD individuals and CAD patients (ie, control/HLP vs. CAD), we evaluated the ranges of L5% and [L5] in individuals with CAD. A *P*-value < 0.05 was considered statistically significant. An odds ratio with a 95% confidence interval was calculated on the basis of the best cut-off point of L5%, [L5], and the LDL/HDL ratio. The Statistical Package for Social Science (version 19.0; IBM SPSS Statistics, Chicago, IL) was used to perform all statistical analyses.
